# Supplementary material for: Robust fractional quantum Hall effect in the N=2 Landau level in bilayer graphene
Source: Nat Commun. 2016 Dec 21;7:13908. doi: 10.1038/ncomms13908 (PMC5187585; doi:10.1038/ncomms13908)
Supplement: Supplementary Information — Supplementary Figures, Supplementary Notes and Supplementary References. [file ncomms13908-s1.pdf]

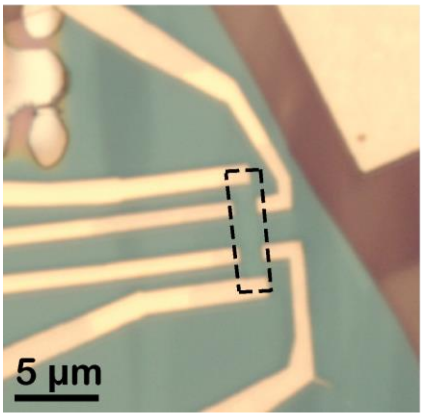

Supplementary Figure 1 | Optical image of device 1. The gold regions correspond to the Ohmic contacts. The dashed lines indicate the boundaries of graphene.

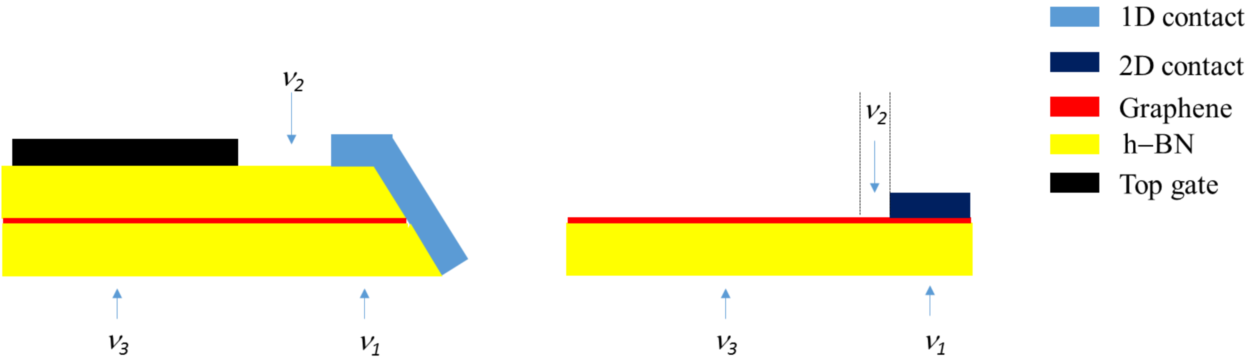

Supplementary Figure 2 | Decision to fabricate open-face bilayer graphene devices. Left panel: A schematic diagram showing regions with different filling factors in a h-BN/graphene/h-BN structure with a one-dimensional contact. Contact-induced doping will make the filling factor near the contact region (ν_1) different from that in the interior that is gated (ν_3) and the intermediate region (ν_2). Right panel: A schematic diagram showing regions with different filing factors in an open-face bilayer graphene device. The graphene region underneath the ohmic contact is at filling factor ν_1. There exists a small region between the dashed lines at filling factor ν_2. The main region of the device is at filling factor ν_3

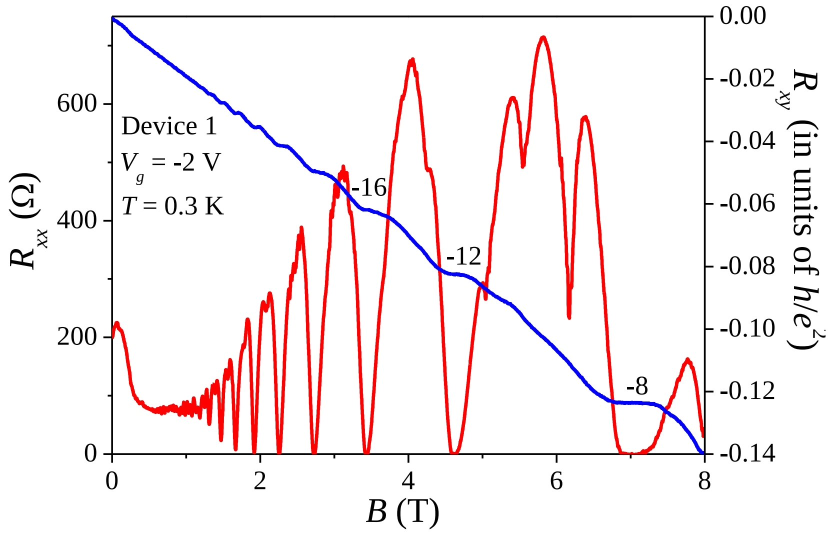

Supplementary Figure 3 | Integer quantum Hall states in device 1. Low-field magnetoresistance measurements on device 1.

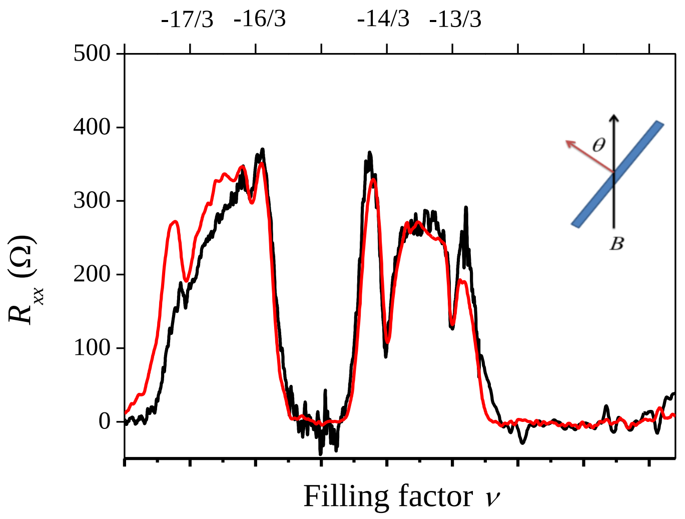

Supplementary Figure 4 | Tilted-field measurements on device 1. Tilted-field measurement for the thirds in the region $4 < |\nu| < 6$. The red curve corresponds to $B_{tot} = B_{\perp} = 25$ T. The black curve corresponds to a tilt angle of $\theta = 56.3^{\circ}$, leading to $B_{tot} = 45$ T and $B_{\perp} = 25$ T).

a

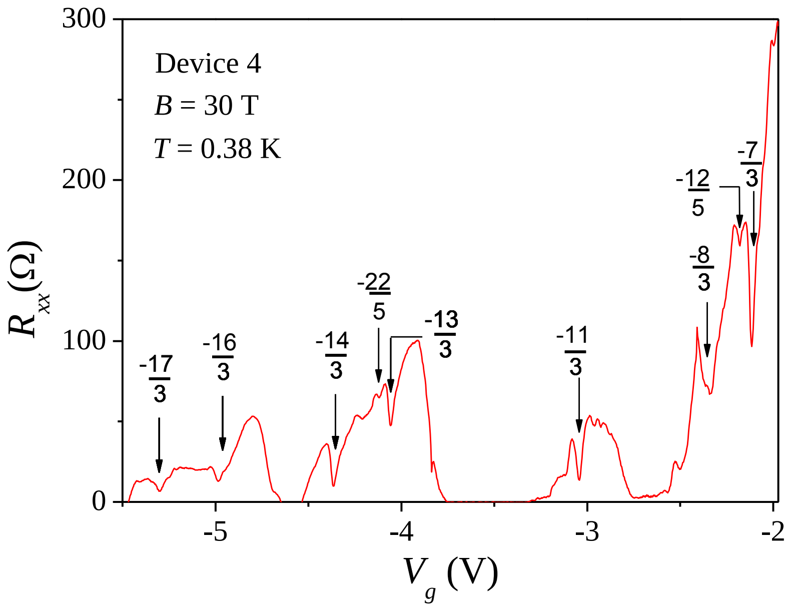

Device 4
B = 30 T
T = 0.38 K
-17/3
-16/3
-14/3
-22/5
-13/3
-11/3
-8/3
-12/5
-7/3
Rxx (Ω)
Vg (V)

b

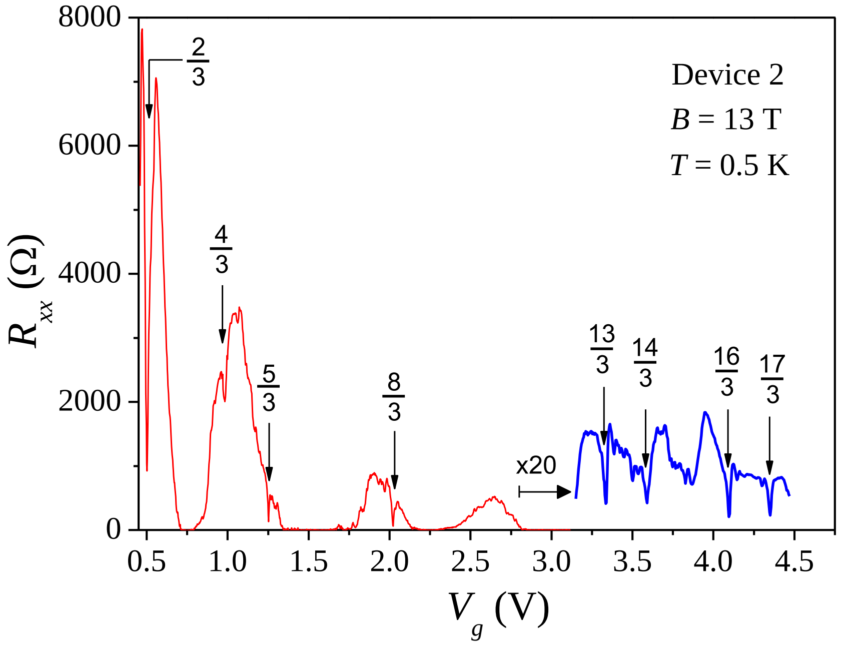

Device 2
B = 13 T
T = 0.5 K
2/3
4/3
5/3
8/3
x20
13/3
14/3
16/3
17/3
Rxx (Ω)
Vg (V)

c

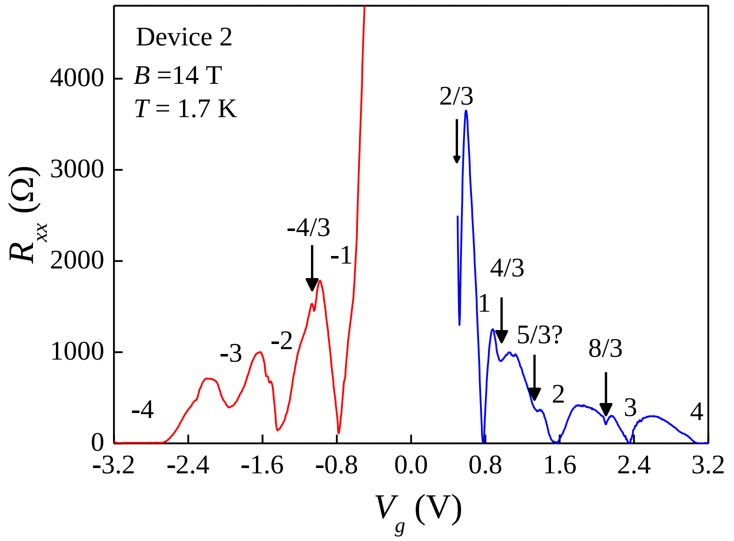

Supplementary Figure 5a, Supplementary Figure 5b and Supplementary Figure 5c | FQH states. Magnetoresistance data showing FQH states in the N=0/N=1 LLs and the N=2 LL.

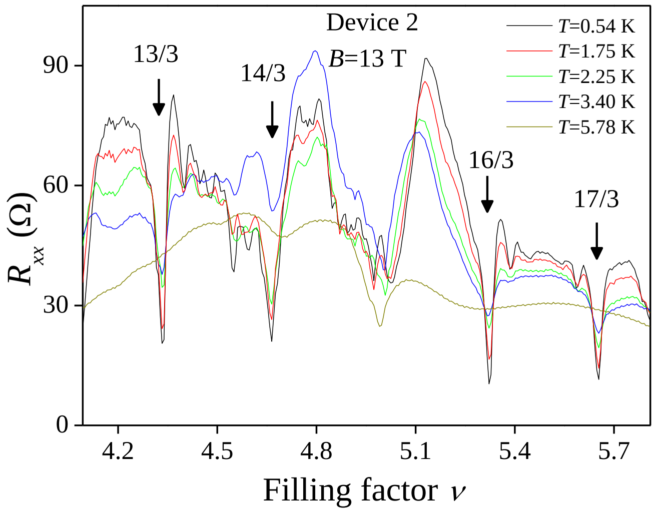

Supplementary Figure 6 | Resonant features and temperature dependence of R_{xx} for 4 < ν < 6. R_{xx} as a function of filling factor for B=13 T at various temperatures (device 2).

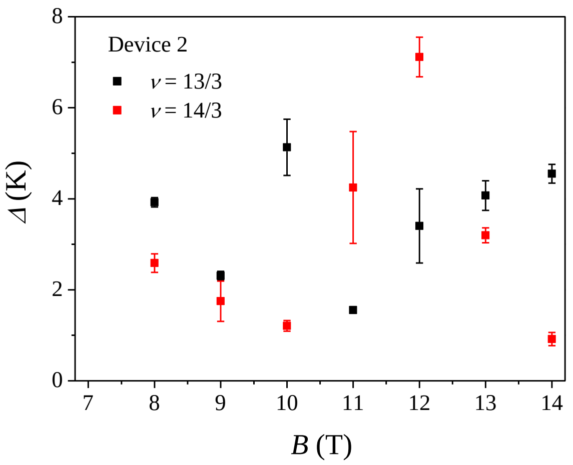

Supplementary Figure 7 | Magnetic field dependence of activation gaps. Measured gap in Kelvin as a function of magnetic field for ν=13/3 and ν=14/3 (device 2). The error bars are due to the error in fitting the data to the Arrhenius law R_{xx} ~ exp[-Δ/(2T)]. See Methods in main paper.

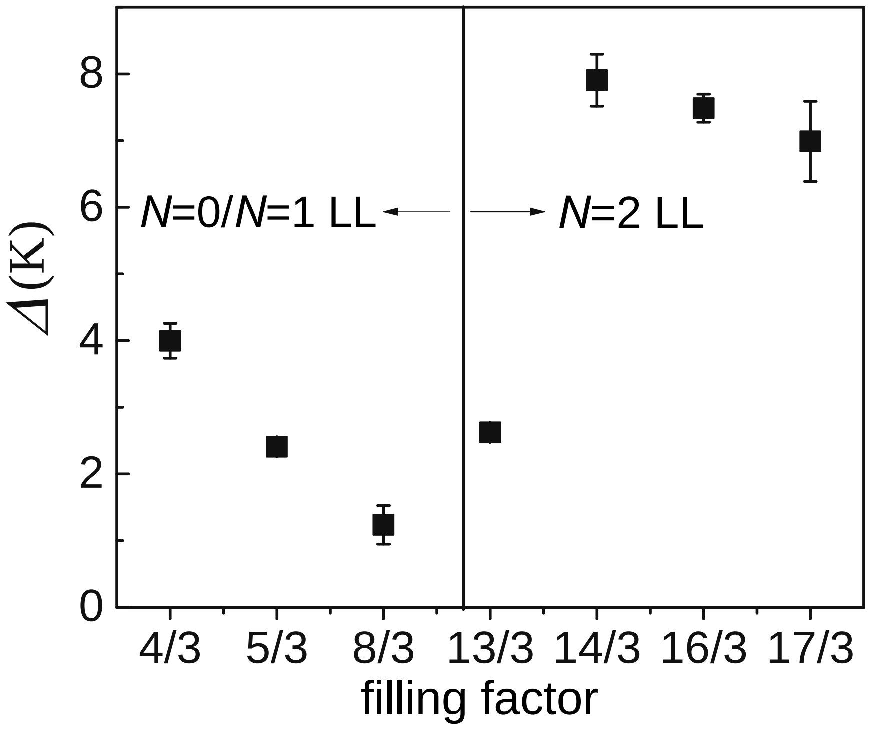

Supplementary Figure 8 | Overview of gaps in the N=0/N=1 LLs and N=2 LL in device 2. The measured activation gaps of the thirds seen in the N=0/N=1 LLs and N=2 LL (device 2). The error bars are due to the error in fitting the data to the Arrhenius law R_{xx} ~ exp[-Δ/(2T)]. See Methods in main paper.

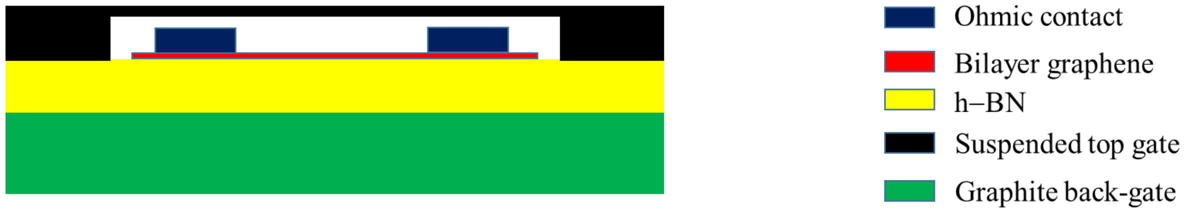

Supplementary Figure 9 | Proposed device for future experiments. A schematic diagram showing a suspended top gated graphite-back gated bilayer graphene device.

Supplementary Note 1 (Fabrication)

For device 1 (Supplementary Figure 1), the calculated field-effect mobility (using the slope of the linear portion of the conductance as a function of gate voltage) was ~200,000 cm^2 $V^{-1}s^{-1}$ at a carrier density 10^{11} cm^{-2}. The devices we measured were strongly insulating at the charge neutrality point at low temperatures, with resistivity above 200 kΩ in some devices at T= 4 K.

The decision to fabricate open-face samples, rather than the recently reported encapsulated fabrication method[1,2], was deliberate. Fully encapsulating graphene in atomically-smooth h-BN layers has led to exceptional cleanliness, as reflected in mobility and mean-free path[1]. Encapsulated devices consist of an h-BN/graphene/h-BN sandwich, with possible additional bottom and top gate (graphite or metal). An encapsulated structure with a top metallic gate was recently used in the study of electric field-induced transitions between fractional quantum Hall (FQH) states in bilayer graphene[2].

Encapsulation requires the formation of one-dimensional metal contacts to the edges of the graphene mesa. Such contacts have proven remarkably robust, but the situation at high field is complicated, as shown in the left panel of Supplementary Figure 2: the deposited metal of such contacts usually extends at least a small distance onto the top of the h-BN layer, above the encapsulated graphene. The graphene sheet is thus exposed to different electrostatic environments in different areas, leading to non-uniform carrier density and, upon application of a perpendicular magnetic field, different filling factors. Underneath the metal contact, the carrier density (with magnetic field, filling factor ν_1) will be determined in part by the differences in the work functions between graphene and the contact metal. A different carrier density (different filling factor, ν_3) will form in the interior of the sample as a result of the electrostatic gating. Between these two areas, a small area of transitional density

(filling factor ν_2) will form. Contacts may be improved by applying a large voltage to the doped Si substrate, to heavily dope the graphene in the contact region with the same polarity as bulk of the film (see Supplementary Material of Ref. 2), but the presence of multiple different filling factors may remain an important complication (see the left panel of Supplementary Figure 2).

In comparison, we plot a schematic diagram of the open-face graphene devices that we fabricated for the FQH measurements in the right panel of Supplementary Figure 2. In the region under the ohmic contact, the filling factor (ν_1) will be different from that in the main region of the device (ν_3). However, since the region underneath the metal contact is highly disordered and can be regarded as a reservoir for carriers, quantum Hall measurements should not be affected. Although there could exist a region with an intermediate filling factor (ν_2) between the dashed lines in an open-face device, the width of this region would be substantially shorter than that in an encapsulated device. Moreover, we note that the Coulomb energy of the FQH states is given by $e^2/(\varepsilon l_B)$. In our open-face devices, the effective dielectric constant is given by the average of the dielectric constants of h-BN and vacuum, which is smaller compared with that in an encapsulated device. This reduction in the effective dielectric constant favors larger FQH gaps in graphene.

Supplementary Note 2 (Integer quantum Hall states)

Supplementary Figure 3 shows a full set of symmetry-broken integer quantum Hall states can be observed at integer filling factors for $B > 5$ T.

Supplementary Note 3 (Tilted-field measurements)

The tilted-field results in Supplementary Figure 4 suggest that the ground states of the thirds seen in the range $4 < |\nu| < 6$ are spin-polarized. At both angles $\theta=0^o$ and $\theta=56.3^o$, the perpendicular magnetic field ($B_\perp$), which sets the energy scale of the Coulomb interaction, is fixed at 25 T. Since the scale of the Zeeman energy is governed by the total magnetic field, which changes from 25 T to 45 T, strengthening of the fractional states would be indicative of the ground-state being spin-unpolarized[3]. With the possible exception of -17/3, which may be slightly weakened, the fractional states are not impacted by the increased Zeeman energy.

Supplementary Note 4 (Resonant structures for $4 < \nu < 5$)

In device 2 the presence of resonant (density- instead of filling-dependent) features for $4 < \nu < 5$, as seen in the Landau fan diagram in Fig. 2d and in Supplementary Figure 6, may prevent accurate quantification of the FQH gaps.

Supplementary Note 5 (Magnetic field dependence of activation gaps)

The magnetic-field dependence of the energy gaps of the thirds we have observed in the range of $4 < |\nu| < 6$ is shown in fig. 3c-d and Supplementary Figure 7. The magnitude of the energy gaps of spinless quasiparticle excitations is expected to vary with field as $\sqrt{B}$. The gaps we measure generally increase with field, though we do not follow them over a sufficient field range to extract a particular power law. The measurable gaps of the four fractional states on which we focus exhibit a linear or slightly superlinear temperature dependence.

Supplementary Note 6 (Overview of FQH gaps)

Supplementary Figure 8 shows that the gaps in the N=2 LL are approximately twice as large as those in the N=0/N=1 LLs. This behaviour is opposite to that seen in GaAs, where the only two known FQH states in the N=2 LL are fragile and experiments at extremely low temperatures are needed to extract meaningful activation gaps[4]. The inverted scale of gaps between N=0 and higher LLs is also seen in monolayer graphene, where robust states (which, however, do not form a complete CF sequence) are seen in the N=1 LL (Ref. 3).

Supplementary Note 7 (Proposed device for future experiments)

The key point of using a suspended top-gate is to make sure that the effective dielectric constant is low, favoring larger FQH gaps. As shown in Supplementary Figure 9, the proposed device allows independent top and back-gate control; thus a large perpendicular electric field between the graphene layer can be produced. Such tunability may allow transitions between different FQH phases and the ability to probe non-Abelian FQH states in both low and high Landau levels.

Supplementary References

1. Wang, L. *et al.* One-dimensional electrical contact to a two-dimensional material. *Science* **342,** 614–617 (2013).
2. Maher, P. *et al.* Tunable fractional quantum Hall phases in bilayer graphene. *Science* **345,** 61–64 (2014).
3. Amet, F. *et al.* Composite fermions and broken symmetries in graphene. *Nat. Commun.* **6,** 5838 (2015).
4. Gervais, G. *et al.* Competition between a Fractional Quantum Hall Liquid and Bubble and Wigner Crystal Phases in the Third Landau Level. *Phys. Rev. Lett.* **93,** 266804 (2004).
